# Perspective on the current state of hyperspectral/multispectral imaging for minimally invasive surgery

**DOI:** 10.1117/1.JBO.31.3.030601

**Published:** 2026-03-20

**Authors:** Mark Witteveen, Cyrille Mooij, Behdad Dashtbozorg, Theo Ruers

**Affiliations:** a Netherlands Cancer Institute, Image-Guided Surgery, Surgical Oncology, Amsterdam, The Netherlands; b University Twente, Department of Nanobiophysics, Enschede, The Netherlands

**Keywords:** hyperspectral imaging, multispectral imaging, minimally invasive surgery, laparoscopy, literature search, bench-to-bedside

## Abstract

**Significance:**

Minimally invasive surgery (MIS) offers substantial benefits to patients, including reduced trauma and faster recovery. However, it limits visual and tactile feedback, which can affect surgical decision-making. Hyperspectral and multispectral imaging (HSI/MSI) are imaging technologies with the potential to provide detailed, real-time tissue characterization, enhancing minimally invasive intraoperative guidance.

**Aim:**

This perspective aims to summarize the current state-of-the-art in the use of HSI/MSI in laparoscopy and endoscopy. It focuses on both technological development and clinical implementation, providing an overview of performance characteristics and translational state.

**Approach:**

A structured literature search was conducted to identify relevant studies. These were analyzed for key system specifications (spectral range, resolution, and acquisition speed) and clinical applications. The studies were further categorized using a “bench-to-bedside” framework to evaluate their level of translational progress.

**Results:**

The majority of studies fall within early translational stages (T1 to T2), with no reports of large-scale clinical trials. Most systems operate in the visible spectrum (450 to 650 nm), optimized for blood-related imaging. Applications include perfusion assessment, nerve visualization using short-wave infrared wavelengths, and tumor detection.

**Conclusions:**

HSI/MSI for MIS is a rapidly developing field with demonstrated potential across a range of applications. Continued research and validation are essential to transition these technologies from experimental use to routine surgical practice.

## 1 Introduction

Hyperspectral imaging (HSI) is a technique that uses the spectral signature from absorption and scattering in diffusely reflected wide-field light to enhance contrast or discriminate changes due to scattering and absorption in a material.^1^^,^^2^ HSI relies on the difference in absorption and scattering within the tissue. When hyperspectral imaging is applied to tissue, chromophores such as blood (oxy- and deoxy-hemoglobin), water, and lipids absorb the light, changing the wavelength-dependent reflection of the light. Moreover, the scattering of light is correlated to changes in the microstructure of the tissue as light scattering is influenced by the size, density, and organization of cellular and extracellular components.^3^^–^^5^ HSI produces 3D data, with two spatial dimensions and one spectral dimension, thereby combining structural information from a material such as shape or texture, with spectral information due to changes in absorption, for example, the vasculature or lipid concentrations.^3^^,^^6^^–^^8^ HSI has many advantages over conventional RGB imaging in applications where tissue identification or discrimination is desired as HSI has both higher discriminating power due to the large wavelength range captured and provides information from deeper tissue layers when combined with near-infrared (NIR, 700 to 1000 nm) and short-wave infrared (SWIR, 1000 to 2500 nm) wavelength ranges.^4^^,^^9^^,^^10^

**Fig. 1:**
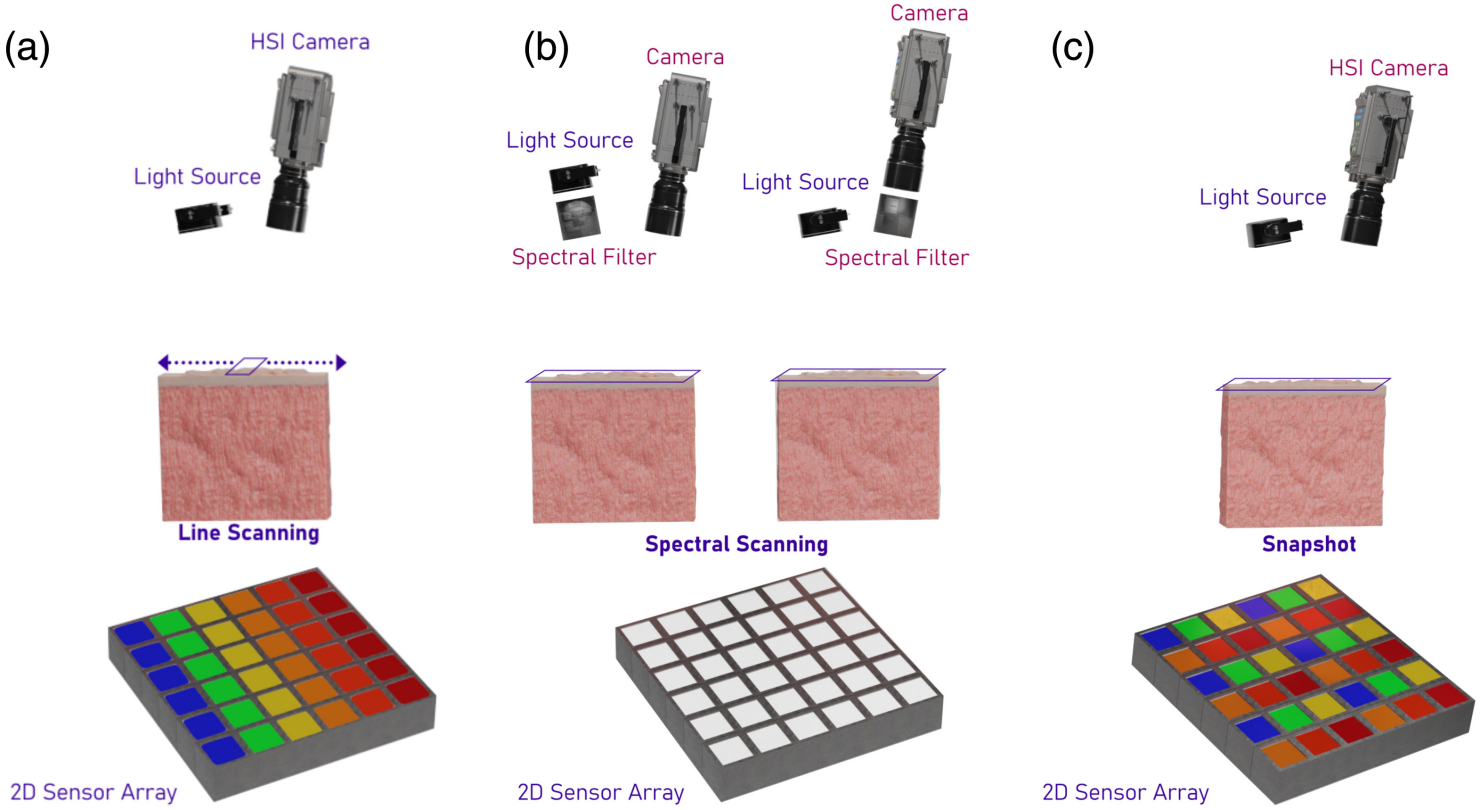
Illustration of the different types of hyperspectral imaging (HSI) commonly used. From left to right: (a) Line-scanning HSI employs an HSI camera that splits light into different wavelengths using a grating or prism, creating a line of spectral data at the surface of the specimen. To capture a full image, either the camera or the specimen must be moved.^11^ (b) Spectral-scanning HSI uses a conventional camera combined with a spectral filter, which can be placed either in front of the camera or the light source. This setup captures an area of the specimen for each wavelength and scans sequentially across wavelengths.^12^ (c) Snapshot HSI involves an HSI camera equipped with a mosaic grid on the sensor that simultaneously captures all wavelengths across a large area, eliminating the need for movement during imaging.^2^

Multiple ways of acquiring HSI can be utilized, with each implementation having advantages and disadvantages.^1^ HSI is often divided into three types: line-scanning, spectral-scanning, and snapshot.^2^ These approaches are shown from left to right in Fig. 1. In a line-scanning hyperspectral camera in Fig. 1(a), the spectral component of the light is projected spatially on the sensor using a grating or prism, where the wavelengths of light are projected along one dimension of a sensor; the second dimension captures a 1D spatial dimension (line). However, due to capturing only one spatial dimension, the line-scanning camera or the sample has to be moved to scan the full surface of the sample. This has the advantage of being able to achieve high spectral and spatial resolution because one dimension on the sensor is being used as a spectral detector, whereas the speed of traverse of the sample or camera determines the spatial resolution; however, this comes at the expense of time due to the movement of the sensor or sample and the setup required for scanning the entire sample surface.^2^

A second type of hyperspectral camera is often referred to as a spectral-scanning camera, where the sensor itself does not distinguish wavelengths but instead a filter mechanism is placed either in the illumination path or the receiving path of the light. This approach is shown in Fig. 1(b), where two types of spectral-scanning setups are shown. The accepted wavelength through the filter is varied to acquire the desired spectral range. Similar to the line-scanning camera, this has the advantage of high spatial and spectral resolution at the expense of time, with acquisition time directly related to the total wavelengths scanned and the type of filter mechanism. However, because the spectral-scanning camera produces a complete image at every wavelength, it is more suitable for applications requiring spatial continuity.^2^ Moreover, as the spectral separation is performed by the filter in-line with either the camera or the light source, the camera can be a conventional camera. However, a spectral-scanning setup does not simultaneously capture spectral and spatial information.

Finally, a third type of hyperspectral camera is a snapshot camera, where the spectral filtering is performed directly on the camera sensor, often in a mosaicking pattern, thus capturing both the spectral and spatial information in a “snapshot.” This approach is shown in Fig. 1(c). Filters are applied over the sensor pixels, capturing the entire spectral range in one acquisition. This has the advantage of speed and compactness but compromises on the spatial and spectral resolution to achieve this. Furthermore, due to the limited size of sensors, the sensitivity of the device is often lower, requiring additional light to illuminate the tissue surface.^13^

Although this diversity has enabled HSI to be explored across many biomedical applications, it has also resulted in substantial heterogeneity in system design, performance metrics, and reported outcomes, particularly in clinically constrained environments such as minimally invasive surgery.

Given these trade-offs in resolution, sensitivity, and acquisition complexity, an alternative approach is to employ multispectral imaging (MSI) techniques. MSI shares many characteristics with hyperspectral imaging but utilizes significantly fewer wavelengths.^1^^,^^2^ This reduction simplifies the acquisition and processing pipeline, often allowing faster imaging while maintaining sufficient spectral contrast for specific diagnostic or intraoperative tasks. When applying MSI, two main strategies are commonly used: either dispersing the bands evenly over a broad spectral range or selecting a limited number of discriminating wavelengths based on prior knowledge or application-specific studies.

A promising use of HSI for medical imaging is within minimally invasive surgery (MIS). MIS has become the standard for many types of surgery over the last years.^14^^–^^16^ MIS has several advantages for the patient: reduced hospital stay, reduced blood loss, and an improved cosmetic outcome after surgery due to reduced incision size.^17^^–^^21^ However, MIS has clear disadvantages for the surgeon: the operation field can only be viewed through a monitor, limiting spatial awareness and depth perception, and tactile feedback is not possible. Furthermore, a reduced field-of-view can further complicate surgery by reducing the three-dimensional context available in open surgery. MIS includes both laparoscopic and robotic surgery, where robotic surgery is increasingly used;^15^^,^^16^ robotic systems are not included in this review due to their closed proprietary architecture. Although MIS has been widely accepted, the technique is still subject to these aforementioned limitations of reduced spatial orientation and absence of tactile feedback.

**Fig. 2:**
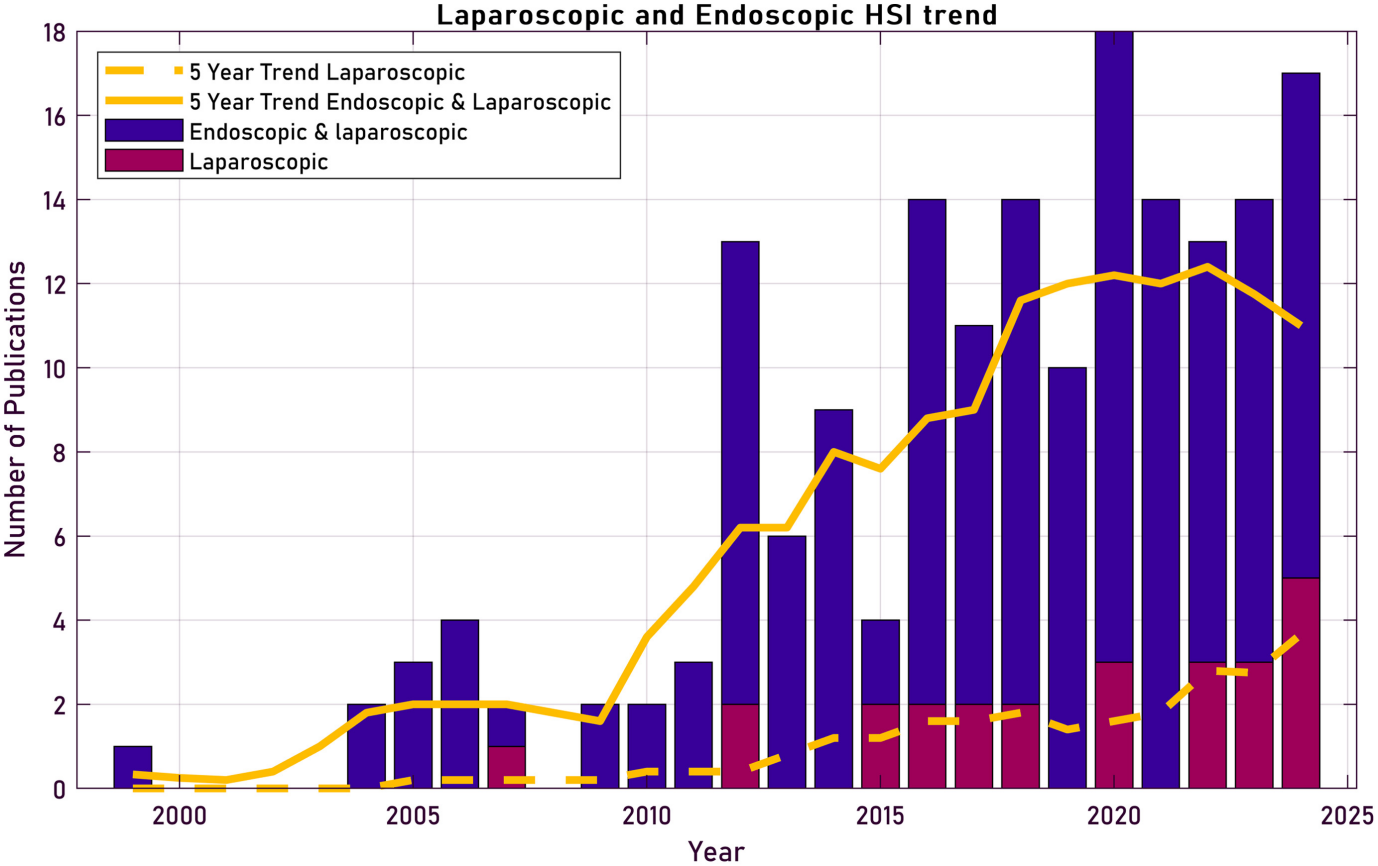
Publication trends for hyperspectral/multispectral imaging in minimally invasive surgery. The first dataset (blue) includes publications related to endoscopy and laparoscopy, whereas the second dataset (purple) excludes endoscopy-related terms. A clear upward trend over the past decade is seen, as indicated by the fitted 5 year trend line for the combination of endoscopic and laparoscopic (solid) and only laparoscopic (dashed), highlighting growing interest in applying hyperspectral/multispectral imaging technologies in minimally invasive surgical procedures.

Several technologies have been developed to compensate for these disadvantages in MIS. Some examples of techniques that have been developed to compensate for these disadvantages: navigated surgical techniques,^22^^–^^27^ and fluorescence imaging.^28^^–^^31^ However, surgical navigation requires additional equipment outside of the laparoscopic equipment, and fluorescence imaging requires the administration of fluorescent dyes that are then used for perfusion imaging or targeted imaging. By contrast, HSI can be combined with laparoscopic imaging seamlessly without the use of external contrast agents to: improve spatial awareness by highlighting certain anatomical structures, to find regions of disease (e.g., cancerous or inflamed regions), or to check perfusion and oxygenation of tissue; aiding the surgeon for improved surgical outcome and reduced surgery time. Similar to laparoscopic surgery, in the field of endoscopy, the use of HSI has the ability to increase contrast and highlight additional anatomical structures. In this literature search, both laparoscopic and endoscopic devices are combined as many of the technological challenges are similar for both applications; because laparoscopic and endoscopic systems share many optical and mechanical constraints, the technological challenges and design trade-offs of hyperspectral and multispectral imaging (HSI/MSI) are largely overlapping for both applications. With the advent of smaller HSI cameras, HSI in combination with laparoscopic surgery has become increasingly popular. The number of papers mentioning the technique has been rising significantly over the last 10 years, as shown in Fig. 2. One of the first papers investigating the use of wavelengths outside the standard visible range for surgery was published by Marcucci et al.^32^ in 1998. A clear increase can be observed first in endoscopic applications, which preceded the use of HSI in laparoscopy. However, since 2015, there has been a notable rise in publications specifically focused on laparoscopic HSI. Despite this growth, the literature remains fragmented, with large variations in acquisition modality, spectral range, performance metrics, and levels of clinical validation.

**Fig. 3:**
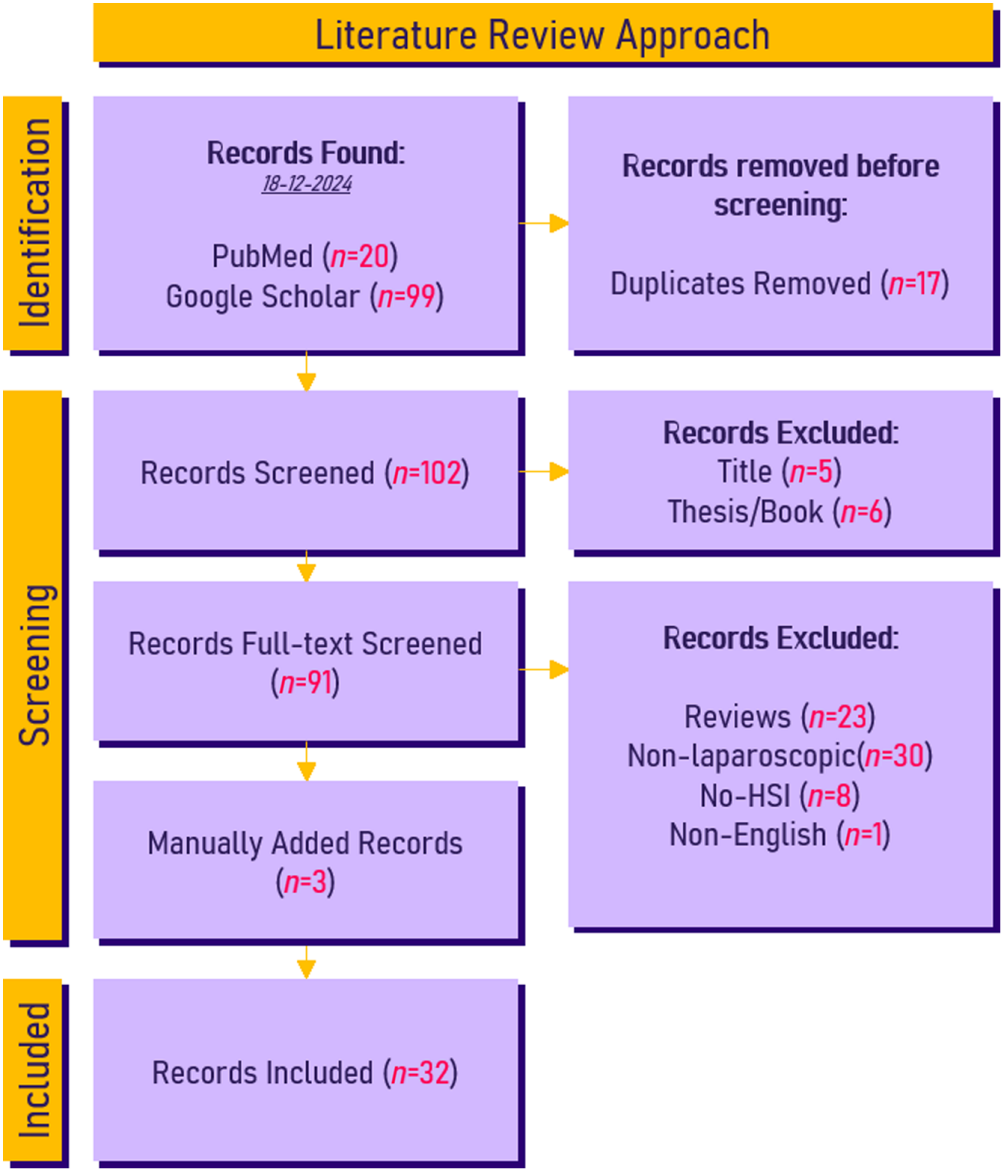
PRISMA flow diagram of the literature search.^33^ Records from PubMed (20) and Google Scholar (99) were included. After duplicates were removed (17), a total of 102 records were screened for eligibility. From these books and theses were excluded (11), leaving 91 full texts to be screened. From these review papers (23), papers not including laparoscopic setups (30), and papers mentioning HSI but not performing diffuse reflection HSI (8) and non-English results (1) were removed. Leaving 32 records included in this overview.

The data underlying Fig. 2 were retrieved from the Web of Science database using predefined queries. For the combined hyperspectral and multispectral studies (blue bars), the following query was used: TS = (“Hyperspectral Imaging” OR “Multispectral Imaging”) AND TS = (“Laparoscopy” OR “Laparoscopes” OR “Minimally Invasive Surgery” OR “Endoscopy” OR “Endoscope”). For the laparoscopic-only studies (purple bars), the query was: TS = (“Hyperspectral Imaging” OR “Multispectral Imaging”) AND TS = (“Laparoscopy” OR “Laparoscopes” OR “Minimally Invasive Surgery”). The number of publications per year was visualized as bar plots, and a 5-year moving average was applied to highlight the overall research trend.

Existing reviews tend to focus either on hyperspectral imaging technology in general or on isolated clinical feasibility studies, yet they do not systematically link device–level specifications to clinical performance and translational readiness. This makes it difficult to assess the maturity of HSI/MSI in the field of MIS and could leave the identification of barriers to widespread adoption obscured. Although prior work has addressed HSI in medical imaging broadly or focused on single applications such as wound assessment using HSI, this is the first comprehensive review that bridges the technology-to-clinic pipeline for HSI/MSI in MIS, addressing gaps such as an integrated assessment of hardware specifications and clinical translation, the role of computational methods and tissue classification, and systematic evaluation of translational readiness to determine where HSI/MSI systems stand relative to clinical translation. Furthermore, it highlights the state of commercial implementation to identify systems that have achieved market development for clinical implementation. Hardware specifications such as spectral range, spatial resolution, and acquisition speed are essential, yet the clinical utility of HSI/MSI ultimately depends on both the hardware as well as the computational algorithms that interpret the spectral data and extract physiologically meaningful information or are able to classify tissues.

The aim of this paper is therefore to provide a structured and up-to-date review of the state-of-the-art of HSI/MSI in laparoscopy and endoscopy, with a particular focus on both technological characteristics and clinical implementation. To achieve this, a systematic literature search was conducted, and the identified studies were analyzed with respect to quantitative and qualitative performance indicators. In addition, the degree of clinical translation is assessed using a bench-to-bedside framework, allowing the maturity of current systems and applications to be compared across studies. By integrating technical specifications, computational methods, reported clinical performance, and translational status, this review clarifies the current position of HSI/MSI in minimally invasive surgery and identifies the key gaps that must be addressed to enable broader clinical adoption.

## 2 Method

### 2.1 Literature Search Strategy

Peer-reviewed articles and conference proceedings focusing on the use of hyperspectral or multispectral imaging technologies in laparoscopy, endoscopy, laparoscopes, and endoscopes were found through a literature search. The primary databases used were PubMed and Google Scholar, as a combination of databases leads to the widest search possible.^34^ The search was implemented in a manner similar to that presented by Saiko et al. (2020),^35^ who performed a literature search for the use of HSI in wound assessment.

The following Boolean search query was used for PubMed and Google Scholar:

(“Hyperspectral Imaging” OR “Multispectral Imaging”) AND (“Laparoscopy” OR “Laparoscopes” OR “Minimally Invasive Surgery” OR “Endoscopy” OR “Endoscope”)

Papers were included from the period between January 2015 and January 2025, to identify only recent advances and the state-of-the-art for HSI; non-English papers were excluded from analysis. For Google Scholar, the first 100 results are considered; after which, the relevancy was deemed too low to include more papers. The PRISMA^33^ flow diagram of the literature search is presented in Fig. 3. From the papers, key points were identified and compared between the included papers, such as technical specifications, implementation state, and key accuracy metrics. Finally, several key papers were manually added from references found in the papers collected by the systematic search, often referred to as “snowballing.”^36^ It is good to mention that a few of the papers found, both through the search criteria and through the snowballing method, discussed HSI/MSI through the use of algorithmic extension or spectral decorrelation of conventional RGB imaging in endoscopy or laparoscopy.^37^^–^^40^ Although these results are interesting and relevant for the intended use of additional spectral discrimination of tissue in MIS, these results do not require any additional hardware and therefore would complicate many of the discussions presented.

### 2.2 State of Implementation Analysis

In this paper, a translational and implementation tiers framework provides a structured approach to describe the development and implementation of medical techniques. This framework is based on several frameworks from the literature^41^^–^^43^; these differ in terminology and stage definitions. The framework presented here is an adaptation tailored to the application of MIS-HSI and does not exactly replicate existing translational models. The framework presented below categorizes progression from basic research through clinical practice:

T1: Preclinical research. This tier focuses on the foundational stages of research, with an emphasis on understanding mechanisms and feasibility. It is further divided into three subcategories:

1. *In silico* (T1.1): Theoretical and computational modelling of biological systems to predict performance and outcomes.
2. *In vitro* (T1.2): Laboratory-based experiments using isolated cells, molecules, or biochemical assays.
3. *Ex vivo* (T1.3): Experiments conducted on tissues or organs outside the organism, in biologically relevant but controlled conditions (e.g., fresh tissue samples, excised organs).

T2: Early clinical research and animal studies. This tier includes studies evaluating MIS-HSI systems in living systems, either in animal models or in limited human settings. It encompasses pilot and feasibility studies as well as first-in-human investigations, including *in vivo* animal experiments. T2 here is adapted to the context of this review and may differ from definitions in the cited references. In addition, *in vivo* studies on animal models are included in this tier as they represent early validation in living systems with feedback from surgical reality.

T3 to T4: Late-stage clinical validation and implementation. These stages conceptually correspond to large-scale clinical trials, regulatory approval, and integration into routine clinical practice. However, it should be noted that no studies identified in the current literature search reach these stages; T3 and T4 are included only for completeness of the framework.

Some results found in the literature search contain research that falls in multiple implementation tiers. If this were the case, the more advanced tier is chosen; e.g., if a part of the paper describes T1.1 *in silico* experiments, which are then verified by T2 *in vivo* clinical trials, the latter will be assigned to the paper as this highlights the advanced state of development of the technology. In addition, besides the implementation tier analysis with the four tiers, the availability for clinicians to acquire a complete MIS-HSI setup is another indication of the implementation state of the technique. As such, when a device is available as a complete commercial device, not as a customized setup that is built from components, it is noted as a commercial device in Table 1.

**Table 1:** Overview of HSI/MSI systems in reviewed papers.

Source	HSI or MSI	Type of HSI/MSI	Commercial device	Light source	Filter system	Camera	Sensor resolution	Bands	Acquisition speed	Specific use
Ayala et al. (2021, 2023)^44^^,^^45^	MSI	Snapshot	No	Xenon	Mosaic filter	XIMEA (MQ022HG-IM-SM4x4-VIS)	272 × 512 px	16	25 Hz	Live perfusion monitoring, ischemia monitoring in laparoscopy
Bali et al. (2024)^46^	HSI	Line Scanning	Yes	LED	—	TIVITA™ Mini	540 × 720 px	100	0.17 Hz	Head and neck tumor surgery
Clancy et al. (2015)^47^	MSI	Spectral-scanning	No	Xenon	LCTF	Thorlabs (DCU223M)	1024 × 768 px	13	0.14 Hz	Intraoperative measurement of oxygen saturation in the intestine
Clancy et al. (2016)^48^	MSI	Spectral-scanning	No	Xenon	LCTF	Thorlabs (DCU223M)	1024 × 768 px	13	0.3 Hz	Organ viability during uterine transplantation in rabbits and sheep
Clancy et al. (2015)^49^	MSI	Spectral-scanning	Yes	Xenon	Filter wheel	SpectroCam	2464 × 2056 px	8	—	Development of a dual multispectral and 3D structured light laparoscope
Fawzy et al. (2015)^50^	MSI	Spectral-scanning	No	LED	LCTF	—	659 × 494 px	6	15 Hz	Detection of early-stage tumors in endoscopy
Fukushima et al. (2024)^51^	HSI	Snapshot	Yes	Xenon	AOTF	CMOS	640 × 480 px	—	—	Hyperspectral imaging for nerve detection
Golubova et al. (2021)^52^	HSI	Line Scanning	Yes	Halogen, Blue LED, Laser	—	Specim IQ CMOS (UI-3360CP-NIR-GL Rev 2)	1280 × 960 px	85^b^	—	Perfusion and metabolism assessment
Han et al. (2016)^53^	HSI	Spectral-scanning	No	Xenon	Filter wheels	Sony (ICX279AL)	582 × 752 px	27	0.24 Hz	Development of a non-contact endoscopic diagnostic support system for colorectal cancer
Hohmann et al. (2017)^54^	MSI	Spectral-scanning	No	Xenon	—	Olympus Video Tower	350 × 370 px	6	0.5 Hz	*In-vivo* multispectral video endoscopy for stomach cancer
Ilgen et al. (2024)^55^	HSI	Line Scanning	Yes	—	TIVITA® Mini	—	540 × 720 px	100	0.2 Hz	Intraoperative hyperspectral imaging for perfusion assessment at anastomotic sites
Jones et al. (2014)^56^	MSI	Spectral-scanning	No	Halogen	Grating	—	1024 × 1024 px	32	0.03 Hz	Hyperspectral imaging for vascular structure visualization
Köhler et al. (2020)^57^	HSI	Line Scanning	No	Xenon/LED	Transmission grating	Sony (IMX290LLR-C, IMX290LQR-C)	1920 × 1080 px	100	0.22 Hz	Laparoscopic system for simultaneous high-resolution video and hyperspectral imaging
Köhler et al. (2022)^58^	HSI	Not specified	No	Xenon/LED	Transmission grating	Sony (IMX290LLR-C, IMX290LQR-C)	1920 × 1080 px	100	—	Comparison of image registration methods
Kumashiro et al. (2016)^59^	HSI	Spectral-scanning	No	LED	Filter wheel (10 filters)	—	800 × 600 px	27	0.2 Hz	Intraoperative oxygenation monitoring
Leitner et al. (2013)^60^	MSI	Spectral-scanning	No	Xenon	AOTF	EMCCD	—	51	0.8 Hz	Detection of cancerous tissue using endoscopy
Lin et al. (2018)^a^	HSI	Line Scanning	Not explicit	Xenon	—	Thorlabs (DCU223M)	1024 × 768 px	270	2 Hz	Reconstruction of tissue surface and hyperspectral imaging
Luthman et al. (2018)^61^	MSI	Snapshot	No	LED	Mosaic filter	CMOS sensors with spectral filters	2048 × 1088 px	41	∼2 Hz	Bimodal reflectance and fluorescence multispectral endoscopy
Ma et al. (2024)^62^	HSI	Line Scanning	Yes	Xenon	Grating	—	1920 × 1080 px	16	—	Enhanced tissue characterization in laparoscopic procedures
More et al. (2016)^63^	HSI	Snapshot	No	LED	Mosaic filter	—	1280 × 720 px	16	0.05 Hz	Multispectral video endoscopy for real-time perfusion monitoring
Pfahl et al. (2022)^64^	HSI	Line Scanning	Partially	Halogen (TIVITA)/LED (HSI-MIS)	—	Mono- chromatic (TIVITA)/RGB + monochromatic (HSI-MIS)	540 × 700 px	100	—	Clinical evaluation of a laparoscopic hyperspectral imaging system
Pfahl et al. (2023)^65^	MSI	Spectral-scanning	No	LED	Narrow-band LEDs	4K color CMOS	960 × 540 px	16	20 Hz	Laparoscopic multispectral system for fast high-resolution perfusion imaging
Pruitt et al. (2023)^66^	HSI	Snapshot	No	—	—	—	—	16/ 15/ 24	120 Hz	Design and validation of a high-speed hyperspectral laparoscopic imaging system
Pruitt et al. (2024)^b^ ^67^	HSI	Snapshot	No	—	—	—	—	29	120 Hz	Design and validation of a high-speed hyperspectral laparoscopic imaging system
Regeling et al. (2016)^68^	HSI	Spectral-scanning	No	—	Mono-chromator	CCD (Axiocam MRm)	1388 × 1040 px	30	—	Hyperspectral imaging with flexible endoscopy for laryngeal cancer detection
Takamatsu et al. (2021)^69^	MSI	Spectral-scanning	No	Halogen	Bandpass filters	—	320 × 256 px	14	—	Tumor detection in *ex-vivo* and *in-vivo* colorectal tumors
Thomaßen et al. (2023)^70^	HSI	Line Scanning	Yes	Halogen (TIVITA)/LED (HSI-MIS)	Mono-chromatic (TIVITA)/RGB + monochromatic (HSI-MIS)	—	540 × 700 px	100	0.14 Hz	*In-vivo* evaluation of a hyperspectral imaging system for minimally invasive surgery
Wisotzky et al. (2020)^71^	MSI	Spectral-scanning	No	Xenon	Filter wheel	CMOS	1920 × 1080 px	6	—	Surgical guidance for the removal of cholesteatoma
Yoon et al. (2019)^72^	HSI	Line Scanning	No	—	Grating	—	—	51	21 Hz	Clinically translatable hyperspectral endoscopy system for the gastrointestinal tract
Yu et al. (2018)^73^	MSI	Spectral-scanning	No	Halogen	LCTF	—	1024 × 768 px	5	—	Hyperspectral imaging for early detection of colorectal cancer
Zhang et al. (2017)^74^	MSI	Spectral-scanning	No	—	Filter wheel	Spectrocam	2464 × 2056 px	8	2.5 Hz	Tissue classification for laparoscopic image interpretation

a The system presented the use of a second camera to upscale to the noted resolution; the slit used has a resolution of 640 pixels.

b To cover the full range, three cameras are used, which are interchanged on the system.

c Approximated from specifications.

### 2.3 Qualitative Assessment Criteria

The qualitative assessment is focused on a few key points. First, the different types of clinical applications are discussed as these dictate the technical specifications that have to be met. Thereafter, the technical specifications of the setup are discussed, with a focus on the type of HSI, the spectral and spatial resolutions. Finally, any found commercial systems are discussed.

### 2.4 Quantitative Assessment Approach

A more quantitative assessment is performed for two components of the found literature. First, for clinical papers, the sensitivity, specificity, and accuracy are discussed. Although these are not directly comparable across all papers, they give an indication of the state-of-the-art performance for three respective applications. Second, any quantitative information on the comparisons between conventional HSI/MSI systems and experimental MIS systems is shown.

## 3 Discussion

This section presents findings from the systematic search and evaluation framework, organized according to a narrative review format. We first present literature search outcomes and system specifications, followed by analysis of spectral algorithms and clinical applications, and conclude with translational status assessment.

### 3.1 System Specifications and Technological Overview

Table 1 shows the publications that utilize an HSI/MSI setup that is directly connected to a laparoscope or endoscope, found through the described literature search. Note that the endoscopic results were manually added.

Multiple ways of acquiring HSI can be utilized, with each implementation having advantages and disadvantages.^1^ HSI is often divided into three types: line-scanning, spectral-scanning, and snapshot.^2^ These approaches are shown from left to right in Fig. 1. In a line-scanning hyperspectral camera [Fig. 1(a)], the spectral component of the light is projected spatially on the sensor using a grating or prism, where the wavelengths of light are projected along one dimension of a sensor; the second dimension captures a 1D spatial dimension (line). However, due to capturing only one spatial dimension, the line-scanning camera or the sample has to be moved to scan the full surface of the sample. This has the advantage of being able to achieve high spectral and spatial resolution because one dimension on the sensor is being used as a spectral detector, whereas the speed of traverse of the sample or camera determines the spatial resolution; however, this comes at the expense of time due to the movement of the sensor or sample and the setup required for scanning the entire sample surface.^2^

A second type of hyperspectral camera is often referred to as a spectral-scanning camera, where the sensor itself does not distinguish wavelengths, but instead, a filter mechanism is placed either in the illumination path or the receiving path of the light. This approach is shown in Fig. 1(b), where two types of spectral-scanning setups are shown. The accepted wavelength through the filter is varied to acquire the desired spectral range. Similar to the line-scanning camera, this has the advantage of high spatial and spectral resolution at the expense of time, with acquisition time directly related to the total wavelengths scanned and the type of filter mechanism. However, because the spectral-scanning camera produces a complete image at every wavelength, it is more suitable for applications requiring spatial continuity.^2^ Moreover, as the spectral separation is performed by the filter in-line with either the camera or the light source, the camera can be a conventional camera. However, a spectral-scanning setup does not simultaneously capture spectral and spatial information.

Finally, a third type of hyperspectral camera is a snapshot camera, where the spectral filtering is performed directly on the camera sensor, often in a mosaicking pattern, thus capturing both the spectral and spatial information in a “snapshot.” This approach is shown in Fig. 1(c). Filters are applied over the sensor pixels, capturing the entire spectral range in one acquisition. This has the advantage of speed and compactness but compromises on the spatial and spectral resolution to achieve this. Furthermore, due to the limited size of sensors, the sensitivity of the device is often lower, requiring additional light to illuminate the tissue surface.^13^

The technical description of the type of systems described in the found literature is shown in Table 1, where it can be seen that a variety of different types of systems are used; each with advantages and disadvantages. The most common type of system is a spectral-scanning system due to the ease of implementation and the versatility such a system brings. These systems often utilize standard machine vision cameras (from vendors such as Thorlabs, New Jersey, United States, or Sony, Minato, Japan) with either a CCD sensor, CMOS sensor, or in the case of Takamatsu et al.^69^ an InGaAs sensor. These spectral scanning setups have an average resolution of 1.2 M pixels and an average of 17 bands. Due to the ability to determine the filter wavelength, five of the spectrally scanning systems are classified as HSI, whereas eight are classified as MSI. However, in spectrally scanning systems, it is often much simpler to change the number of wavelengths compared with the other types of systems, making it possible to convert an HSI system into an MSI by selecting fewer bands. This is reflected in the number of bands the spectrally scanning systems utilize, with an average of 17 bands, with some spectral-scanning systems having as many as 51, whereas others have as few as six bands.

Unlike spectral-scanning systems, line-scanning systems offer high spectral resolution by capturing one spatial line at a time while scanning across the target. These systems in general are predominantly HSI, with eight of the identified systems falling into this category and only two being classified as MSI. The included papers have an average resolution of 1.0 M pixels and an average of 95 spectral bands, which is significantly higher than both the spectral-scanning and the snapshot cameras. Line-scanning systems are often equipped with grating-based spectral dispersion elements, allowing them to cover a broad wavelength range with fine spectral sampling, reflected in the average of 95 spectral bands in the found systems. Compared with snapshot or spectral-scanning systems, line-scanning setups require precise motion control but provide superior spectral resolution. The studies utilize different approaches to move the scanner to be able to move the imaging area across the tissue. Some systems discussed have an internal scanning system, reducing the need for fine movement by the operator.

Snapshot systems were found to be the least common type, favored for their ability to capture spectral data in a single exposure, making them ideal for imaging applications that require acquisitions within the timescale of surgery. However, they have limited spectral bands compared with line-scanning and reduced resolution compared with spectral-scanning systems. These systems typically employ CMOS sensors with mosaic filters, achieving an average resolution of 0.9 M pixels and 22 spectral bands. Of the snapshot systems, six are HSI, whereas two are MSI, reflecting the tendency of these systems to be optimized for hyperspectral imaging despite their limited flexibility in spectral sampling. Although snapshot systems offer significant speed advantages, they often trade spectral resolution and flexibility for this increase in acquisition speed as the number of bands is fixed by the filter design. Furthermore, compared with the spectral-scanning systems, utilizing application-specific wavelength bands could be expensive to achieve with snapshot cameras due to the need for a custom sensor.

**Fig. 4:**
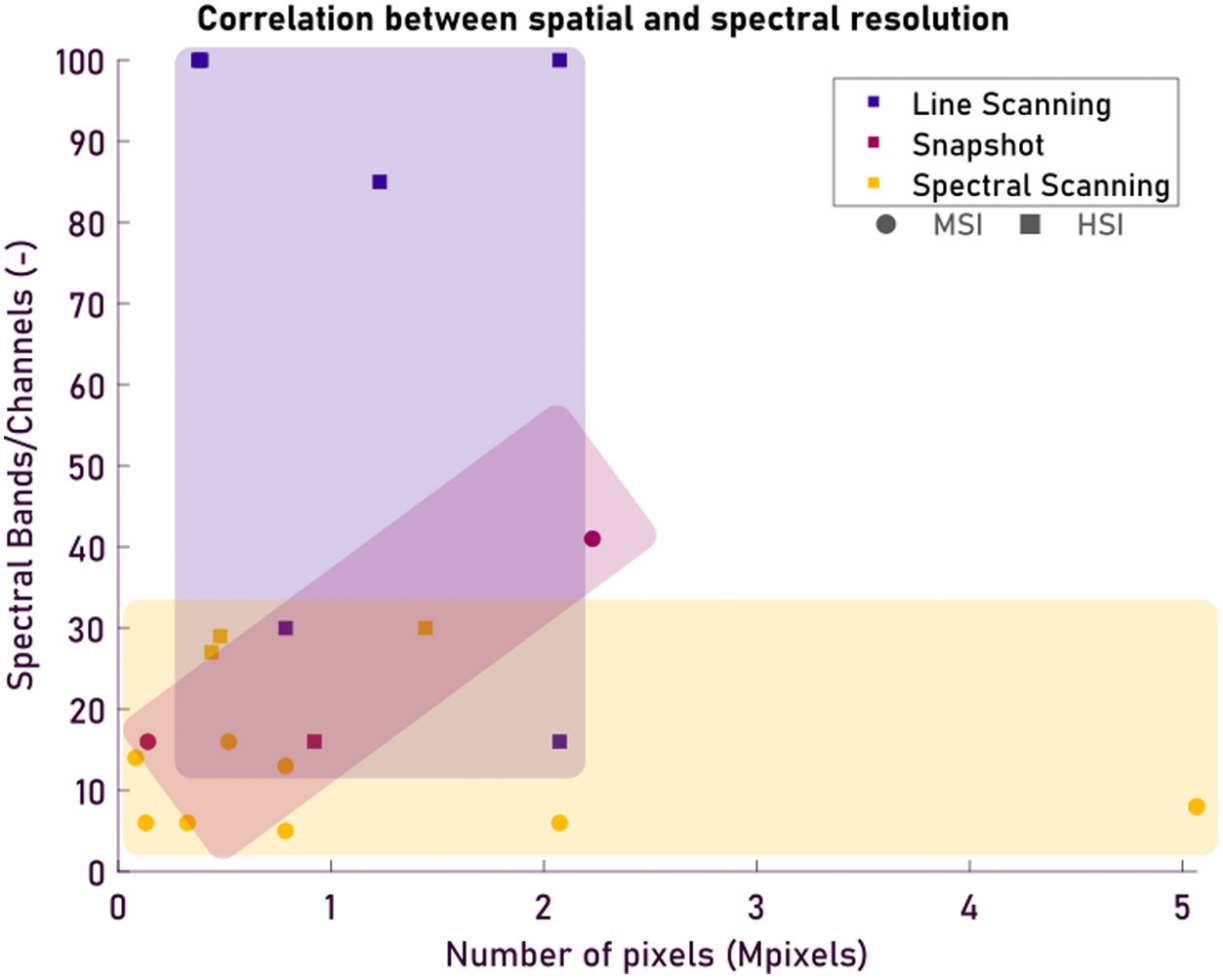
Scatter plot showing the correlation between spatial and spectral resolution in the found hyperspectral imaging systems discussed in Tables 1 and 2. The x-axis represents the spatial resolution in megapixels (Mpixels), whereas the y-axis shows the number of spectral bands or channels. Different acquisition strategies are color-coded: line scanning (blue), snapshot (purple), and spectral-scanning (yellow). Finally, HSI is indicated with a square and MSI with a circle marker in the figure.

In Fig. 4, the trade-off for line scanning and spectral-scanning systems can be seen. Line scanning systems typically achieve high spectral resolution, with a maximum of 100 bands in the found literature, but with a reduced spatial resolution. Spectral-scanning systems are capable of higher spatial resolutions, with a max of 5 Mpixels; however, most have a reduced spectral resolution due to the increased time and complexity that comes with the increased spectral bands. Finally, snapshot systems exist mostly between the line scanning and spectral-scanning systems, having chosen a medium point between spatial and spectral resolution as they are designed to work for multiple wavelengths. These effects are visible in Fig. 4, where the three types of HSI show clear domains in either spectral (line-scanning), spatial (spectral-scanning), and in-between (snapshot).

**Table 2:** Overview of selected studies with corresponding wavelength ranges and bands.

Study	Min wavelength (nm)	Max wavelength (nm)	Bands
Ayala et al. (2021)/(2023)	465	641	16
Bali et al. (2024)	500	1000	100
Clancy et al. (2015)/(2016)	500	620	13
Clancy et al. (2015)	425	775	8
Fawzy et al. (2015)	400	760	6
Fukushima et al. (2024)	500	620	—
Golubova et al. (2021)	400	700	85
Han et al. (2016)	405	665	27
Hohmann et al. (2017)	400	650	6
Ilgen et al. (2024)	500	995	100
Jones et al. (2014)	400	720	32
Köhler et al. (2020)/(2022)	500	1000	100
Kumashiro et al. (2016)	420	700	29
Leitner et al. (2013)	400	650	10
Lin et al. (2018)	460	690	30
Luthman et al. (2018)	463	891	41
More et al. (2016)	480	680	16
Ma et al. (2024)	460	600	16
Pfahl et al. (2022)	500	1000	100
Pfahl et al. (2023)	404	957	16
Pruitt et al. (2023)	460	600	16
Pruitt et al. (2023)	600	870	15
Pruitt et al. (2023)	660	960	24
Pruitt et al. (2024)	460	850	29
Regeling et al. (2016)	390	680	30
Takamatsu et al. (2021)	1000	1400	14
Thomaßen et al. (2023)	500	1000	100
Wisotzky et al. (2020)	250	800	6
Yoon et al. (2019)	450	750	51
Yu et al. (2018)	850	850	5
Zhang et al. (2017)	470	700	8

For surgical applications, the ability to capture real-time data without interrupting the surgery would be one of the most important factors for successful implementation, thereby complicating the use of line-scanning and spectrally scanning systems. As shown in Table 1, line-scanning and spectral-scanning systems typically operate at sub-Hz acquisition speeds (often below 1 Hz) as their acquisition time scales with either mechanical scanning or sequential wavelength selection, making them less suitable for continuous video-rate guidance. However, if the HSI laparoscope is the only used laparoscopic system in the surgery, the spatial resolution has to be comparable to conventional surgery, reducing the implementability of low-resolution snapshot cameras. Snapshot systems, by contrast, achieve the highest acquisition speeds, ranging from several Hz up to true video-rate operation (25 to 120 Hz) because all spectral information is captured simultaneously; but this comes at the expense of reduced spatial or spectral resolution. Either specially designed low-band spectral-scanning systems or high-resolution snapshot cameras might be most suited for clinical applications, with the final system choice reflecting a trade-off between acquisition speed, spatial fidelity, and spectral content dictated by the intended clinical task.

From Table 1, it can also be seen that there are a number of commercial systems that have been developed and are being used. The most common is the TIVITA^®^ Tissue system, both the open system and the MIS system, which is a fast system operating between 500 and 1000 nm with 100 bands. This system produces a number of parameter maps, which represent the blood volume, blood saturation, water, and lipid concentrations. Some papers then use these maps to further discriminate between tissue states or types.^55^^,^^75^ Another commercial system that is represented in Table 1 is the Specim IQ HSI system, which is a handheld system that was adapted to be used in combination with a laparoscope.^52^ Finally, the SpectraCam is another commercial system that is utilized by two papers; this is a filter-wheel system utilizing 8 bands with customizable filters.^47^^,^^74^ The advantage of this system is the ability to use custom filters to tailor the exact bands to the needed wavelengths for the specific application.

Beyond the detector modality itself, the reviewed systems also differ substantially in how HSI is physically integrated into the surgical environment, with architectural design choices directly influencing usability, performance, and clinical feasibility.

The practical realization of HSI in the operating room relies on a few key architectural choices: (i) the mechanical coupling of the detector to the scope through relay optics, (ii) the selection of the illumination source, and (iii) the choice of rigid versus flexible endoscopes. These decisions shape system cost, ergonomics, compatibility with existing surgical workflows, and the likelihood of regulatory approval. The dominant strategy across the literature is using relay optics with an external camera. In this configuration, a dedicated line-scanning, spectral-scanning, or snapshot HSI detector is linked to the scope’s proximal side. This design offers modularity, permitting the use of any conventional scope with no or minimal internal modification, and allows the detector to be upgraded independently. The main drawback is the higher (10% to 30%) transmission loss and added weight at the proximal side of the device compared with chip-on-tip designs. Furthermore, the choice of a conventional laparoscope would make it easier to introduce an HSI/MSI system; however, depending on the transmission of the laparoscope and the desired wavelengths, ranges might not be suited. As laparoscopes are produced for conventional RGB imaging, wavelengths below the visual range (λ<450  nm) or above (λ>750  nm).

**Fig. 5:**
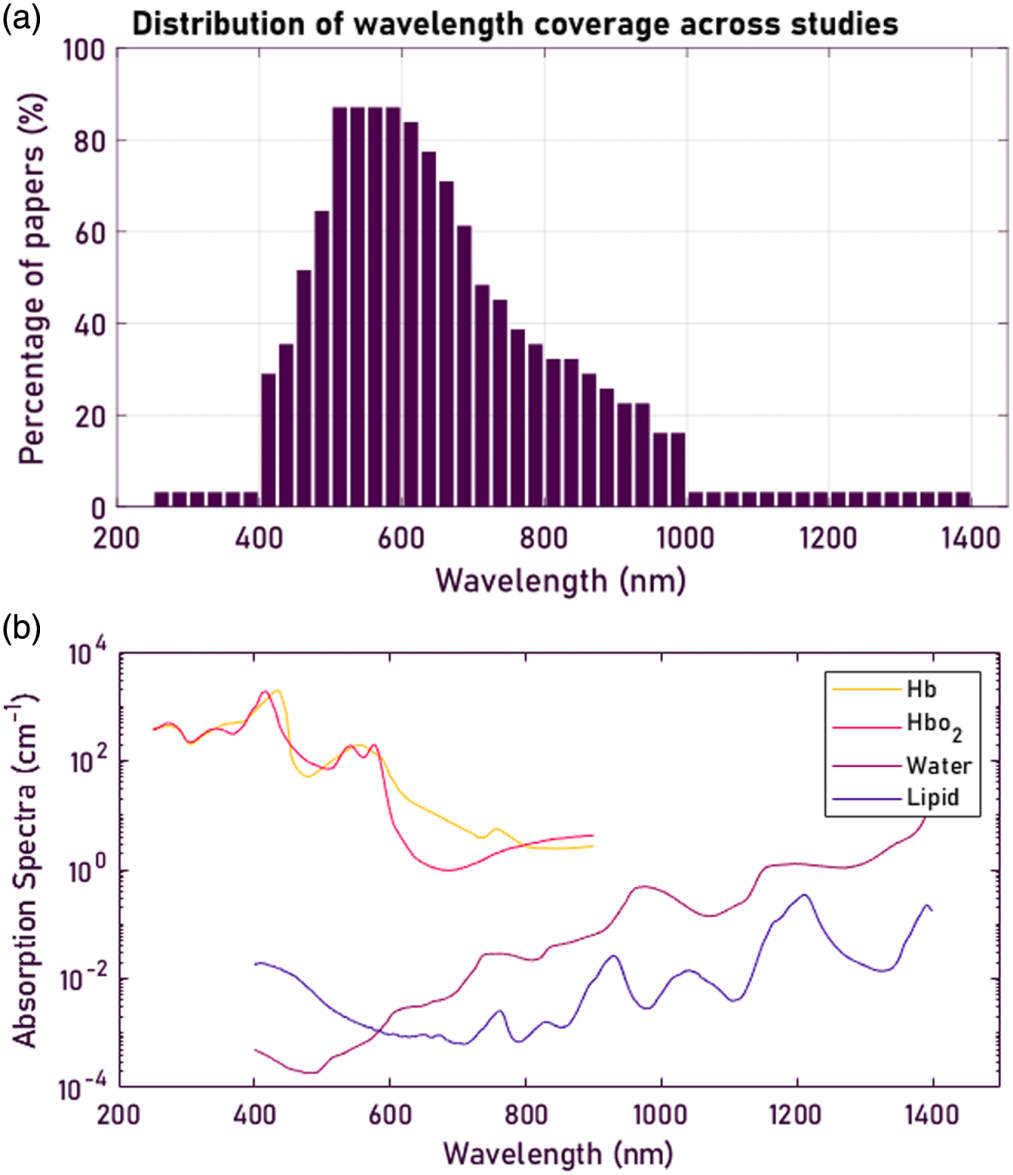
(a) Distribution of wavelength coverage across the papers, with the majority of papers covering between 400 and 1000 nm. (b) Chromophores in biological tissue, showing a clear overlap between the wavelengths covered by the papers and discriminating wavelengths between the chromophores.^3^ Note that scattering is not shown but is also a major contribution to the discrimination between tissue types.

A large number of papers favor Xenon arc lamps for their broad, continuous, and relatively flat spectrum (350 to 1000 nm) and high photon flux, enabling fast acquisition but generating substantial heat and requiring large cooling assemblies. Halogen lamps, used more sparingly, provide continuous broadband illumination with lower intensity in the VIS but higher output in the NIR and SWIR wavelengths while still generating significant heat and exhibiting temperature-dependent spectral output. Over the most recent decade, a shift toward LED arrays can be observed, reflecting their wavelength-selective illumination, compact form factor, lower thermal load, and reduced cost compared with both halogen and xenon sources. All of these ranges match the trend of the concentration of employed wavelength bands in the 400 to 700 nm range, with extension toward 1000 nm observed in Fig. 5. A wavelength above 1000 nm, as in Takamatsu et al.,^69^ needs halogen lights, and wavelengths below 400 nm need Xenon sources, as in Wisotzky et al.^71^

Rigid and flexible endoscopes each offer advantages that make them suitable for different clinical applications, and their suitability largely dictates the technological requirements for integrating HSI. Rigid scopes, favored in procedures with direct access such as laparoscopy or arthroscopy, provide straight optical paths that support high numerical aperture, stable calibration, and minimal light loss. These characteristics simplify relay-optics design and enable reliable internal scanning mechanisms (e.g., rotating mirrors). Flexible scopes, by contrast, are essential for navigating anatomically complex regions such as the gastrointestinal or respiratory tracts; however, their ability to bend introduces challenges, including reduced light transmission and shifting imaging planes, complicating spectral calibration.

There is no single best HSI implementation; the choice must be dictated by the clinical task: use line-scanning when maximal spectral fidelity is essential (accepting slower, scan-based acquisition, and precise motion control); use spectral-scanning when high spatial resolution and flexible wavelength selection are required (at the cost of longer multiwavelength acquisition times); and use snapshot HSI when real-time, video-rate guidance is the priority (accepting reduced spectral flexibility and usually lower sensitivity). Furthermore, the literature shows external-camera systems with relay optics are the most practical near-term route (chip-on-tip solutions are not represented), and LED-based illumination is increasingly preferred for its spectral selectivity, compactness, and lower thermal load. For laparoscopic clinical translation specifically, a modular external-camera architecture combined with either application-targeted low-band spectral-scanning or a high-resolution snapshot design, deployed on a rigid scope with well-characterized relay optics and LED illumination, represents the best balance of image quality, intraoperative usability, and regulatory practicality.

### 3.2 Calibration and Processing Analysis

For clinical applications of HSI in surgical practice, the robustness and accuracy of any developed algorithms are key factors for successful implementation. One of the steps that needs to be discussed is the spectral calibration performed for the included papers. In addition, if performed, any pre-processing steps are also key for the implementation of the technique in surgical practice. Finally, key computational elements should be discussed, including algorithms for tissue classification, methods for extracting or interpreting physiological parameters (e.g., linear regression, deep learning, and spectral unmixing), and the need for real-time spectral processing, which is critical for clinical translation.

#### 3.2.1 Spectral calibration

Spectral calibration is essential for correcting instrument-dependent variations and converting raw sensor measurements into meaningful reflectance spectra. One of the most commonly used methods is by taking a white (reference) and black (dark) measurement as:^44^^,^^46^^,^^47^^,^^51^^,^^53^^,^^57^^,^^60^^,^^62^^,^^65^^,^^69^^,^^71^^,^^72^^,^^76^^,^^77^

$$I_{\text{calibrated}}=\frac{I_{\text{measured}}-I_{\text{black}}}{I_{\text{white}}-I_{\text{dark}}},$$ (1)

where $I_{\text{measured}}$ is the raw recorded intensity, $I_{\text{black}}$ (or dark reference) represents the sensor signal acquired with the illumination source turned off, and $I_{\text{white}}$ is the intensity measured from a high–reflectance white standard (typically Spectralon). This normalization corrects for illumination spectrum, sensor-specific sensitivity, and electronic noise, ensuring that the resulting calibrated reflectance values are comparable across wavelengths, measurements, and acquisition conditions.^78^ Some perform only part, either dark correction or white calibration, of the method mentioned in Eq. (1).^49^^,^^50^^,^^54^^,^^59^^,^^61^^,^^63^^,^^66^^,^^67^^,^^79^^,^^80^ Fawzy et al.^50^ presented an alternative calibration, based on Eq. (1), wherein they introduced a factor $k_{i}$, which they refer to as a geometry correction factor for distance and angle of the laparoscope, where they estimate $k_{i}$ for every measurement separately. This geometric correction factor approach is utilized in a small number of papers.^50^^,^^59^^,^^66^

To categorize the spectral accuracy of their system, some papers use a spectral line verification method. Yoon et al.^72^ used a combination of mercury and neon argon lamps to verify their spectral reflectance captured. Furthermore, a verification with a spectral line could be used to correct the measured signal; Kohler et al.^57^ used a krypton lamp to calibrate their device once, after which they used the calibration in Eq. (1).

#### 3.2.2 Pre-processing or normalization

Pre-processing aims to improve image quality and suppress artifacts, both spatial and spectral, using algorithmic means. The most common pre-processing that is applied is spectral pre-processing (normalization), where the spectra are processed to reduce the effects of varying distance, glare, and nontissue-specific variations.^81^ Standard normal variate (SNV) was found to do a good job of reducing the effect of glare and distance, which is a key factor influencing the spectra when applying HSI/MSI in MIS.^46^^,^^69^^,^^81^ In addition, SNV $l_{1}$ and $l_{2}$ normalization approaches were also applied to reduce nontissue-specific variations.^44^^,^^45^^,^^61^

When fiber-based scopes are utilized for the HSI prototype, a type of spatial pre-processing has to be applied to remove the lines between the fibers, often referred to as a “honeycomb pattern.”^50^^,^^53^^,^^61^^,^^72^^,^^77^^,^^82^^,^^83^ These de-pixelations or corrections for the honeycomb pattern are done mostly using Fourier filtering.^61^^,^^72^^,^^83^ Furthermore, some papers analyzed deformation in the imaging system for pincushion or barrel distortion,^54^^,^^61^^,^^62^ and some correct for this.^56^^,^^71^^,^^72^ This is important when matching the HSI imaging to other modalities or for the correction of highly deformed images for visualization for surgeons.

Several papers also perform some image registration or motion compensation.^44^^,^^56^^–^^58^^,^^60^^,^^70^^,^^72^^,^^79^ Most papers do this algorithmically, where they use some feature identification method (SIFT, FAST, BRISK, optical flow) and track points either between wavelengths with a spectral scanning device, or between lines in a line-scanning device. This is key for MIS use of HSI for systems that do not capture at video-rate; snapshot systems, however, often do not need this approach as they capture all bands simultaneously.

#### 3.2.3 Parameter extraction and processing

After calibration and pre-processing, feature-extraction methods convert spectral data into clinically interpretable parameters. The two most commonly applied metrics are: to convert the HSI data into tissue parameters (saturation, blood indices, and water indices), which are directly interpretable by clinicians;^47^^–^^50^^,^^52^^,^^55^^–^^58^^,^^61^^,^^64^^,^^65^^,^^70^^,^^72^^,^^75^ or by processing the HSI data into segmentation masks or classifications depending on what the goal of the paper is.^44^^–^^46^^,^^51^^,^^53^^,^^54^^,^^59^^,^^60^^,^^68^^,^^69^^,^^71^^–^^74^^,^^77^ An example of the first method is used by the TIVITA® Tissue system and the TIVITA® MIS system,^46^^,^^55^^,^^70^^,^^75^ Ilgen et al. (2024)^55^ used this to assess the quality of anastomosis in esophageal surgery using the near-infrared perfusion index (NIR-PI), which is an index that describes the perfusion of deeper layers of tissue.^84^

Newer papers tend to utilize deep learning (DL) approaches for a variety of different applications.^44^^,^^45^^,^^51^^,^^52^^,^^58^^,^^62^^,^^69^^,^^77^^,^^85^ The most apparent and straightforward applications of DL techniques are in a classification approach, tissue classification, and tumor or nerve identification leverage neural networks to distinguish pathological and anatomical structures based on spectral signatures.^51^^,^^69^ Similarly, ischemia detection and perfusion monitoring, deep learning enables real-time detection of abnormal tissue spectra and reconstruction of physiological maps, similar to the TIVITA® Tissue system maps discussed earlier.^45^^,^^52^ Finally, for specific laparoscopic designs where there are multiple sensor data sources, tracking, or image registration is needed. To achieve this tracking and image registration, CNN-based methods can perform ROI tracking and elastic registration of laparoscopic video and spectral data.^44^^,^^45^^,^^58^ Similarly, super-resolution and spectral reconstruction, CNN and U-Net architectures fuse RGB and hyperspectral data to recover high-resolution spectral information.^62^^,^^77^

### 3.3 State of Implementation Analysis and Clinical Translation

**Fig. 6:**
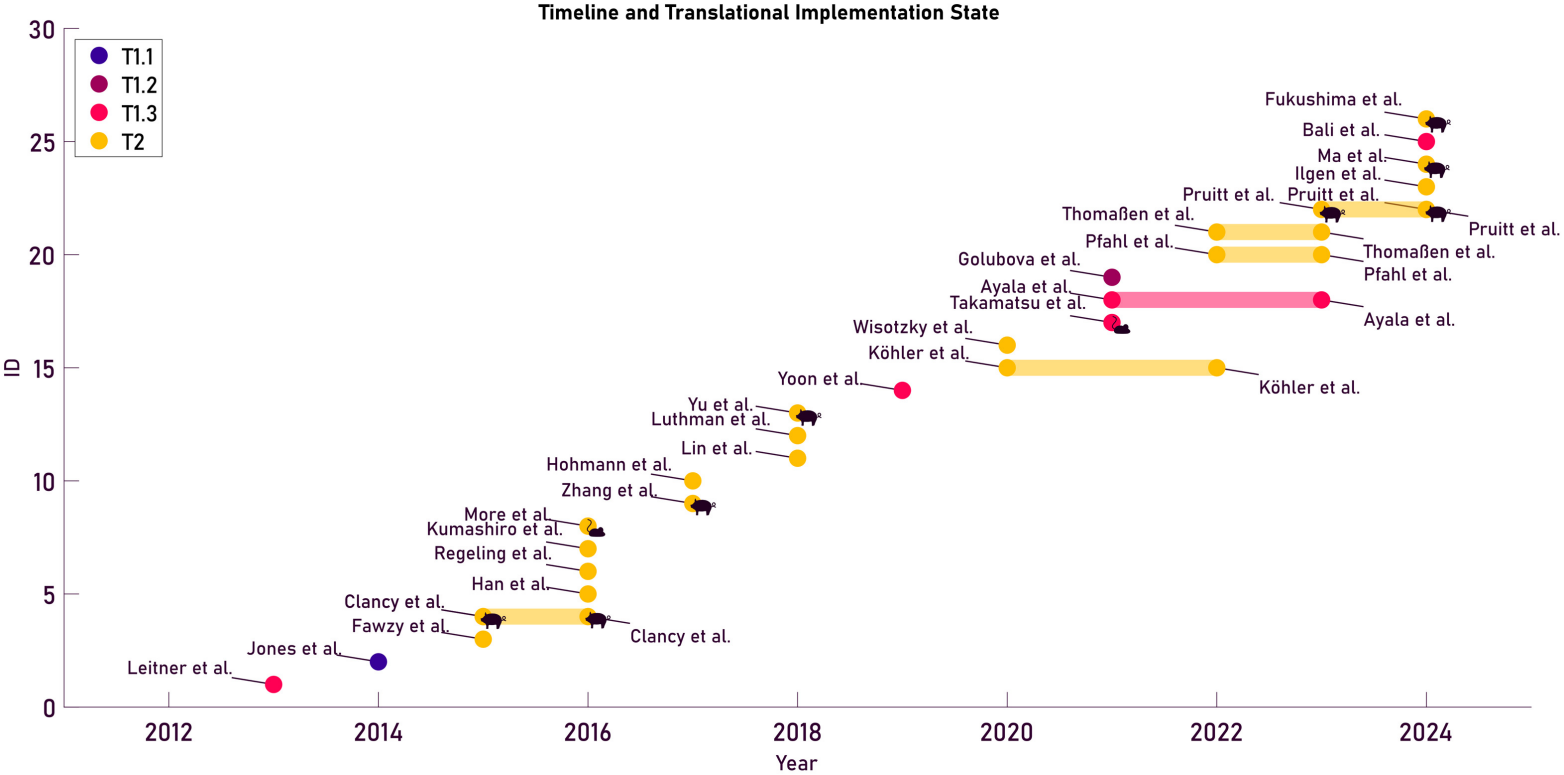
State of implementation of the found literature over time, with authors who published multiple times being connected in the figure. The translation implementation tiers are shown from left to right, with additional distinctions in tier 1 (T1 between: *in silico* (T1.1), *in vitro* (T1.2), and *ex vivo* (T1.3). Note that when a study concerns an animal study, this is indicated in the figure with a small icon. Finally, follow-up studies by the same author are indicated with a line between these studies.

As discussed in the Sec. 2.2, different states of implementation can be identified using a “bench-to-bedside” framework. These different states of implementation are shown in Fig. 6, using the translational and implementation tiers framework, which progresses from preclinical research (bench) to clinical application (bedside). All of the identified studies fall under T1: preclinical research or T2: early clinical studies, focusing on fundamental mechanisms, feasibility, and early clinical implementation.

It is important to note that more early translational stages are expected in emerging technologies; however, the absence of T3 to T4 studies gives an important insight into the implementability of these technologies. Unlike pharmaceuticals or devices with established regulatory pathways, HSI/MSI in MIS faces unique barriers to late-stage clinical translation due to a combination of: difficult regulatory pathways for novel imaging modalities, the lack of agreed-upon clinical endpoints or standardized performance metrics across studies, and due to the fairly recent (2015) development of HSI in MIS the technical maturity is still in development for the technology.

With regard to the T1: preclinical research, most studies focused on *ex vivo* research in humans or animal models. Leitner et al. (2013)^60^ and Jones et al. (2015)^56^ highlighted the wide range of research within the T1 stage of research. With Leitner presenting a multispectral video-endoscopy system validated on *ex vivo* tissue biopsies, and Jones describing compensation techniques for deblurring images due to motion that could be introduced during *in vivo* measurements. Several studies are on the interface between preclinical and early clinical applications, combining T1: preclinical research and T2: early clinical studies. For instance, Fawzy et al. (2015)^50^ introduced a multispectral endoscopic imaging system, initially validated on standards (T1) and subsequently tested on 10 patients (T2). Similarly, Yoon et al. (2019)^72^ presented a clinically translatable hyperspectral endoscopy system with *ex vivo* measurements on chicken and human tissues (T1) and demonstrated its potential for clinical application (T2).

In the category of T2: early clinical studies, studies primarily focused on evaluating new methods in humans, emphasizing feasibility and safety. Ayala et al. (2023)^45^ developed a personalized approach for ischaemia detection using MSI, validating their proposed ischaemia index in a patient study with 10 participants undergoing partial nephrectomy. Bali et al. (2024)^46^ conducted a feasibility study involving 12 head and neck cancer patients, optimizing the workflow for real-time tumor classification using endoscopic HSI. Han et al. (2016)^53^ used HSI *in vivo* to differentiate malignant colorectal tumors from normal tissue, contributing to the development of diagnostic systems. Ilgen et al. (2024)^55^ evaluated the TIVITA™ Mini HSI system during minimally invasive esophagectomy, focusing on tissue perfusion and feasibility in a clinical setting. Last, Thomaßen et al. (2023)^70^ analyzed 19 patients with an HSI camera for minimally invasive surgery, TIVITA™ Mini, comparing it with open surgery systems to evaluate its clinical potential.

Finally, from the figure, it can also be seen that no studies were found in this analysis that exceed a T2: early clinical studies state of implementation. However, some devices discussed in these papers seem ready to translate HSI from T2: early clinical studies to T3: late-stage clinical studies as they present good results using CE-approved devices.^45^^,^^55^^,^^70^

### 3.4 Clinical Applications and Performance Metrics

From the found literature, it can be seen that a variety of applications are discussed within surgical and diagnostic procedures. In the following section, we discuss those with explicitly described clinical applications.

One significant application area is real-time monitoring of perfusion and ischaemia during surgeries. By measuring oxygen saturation and blood volume in tissues, surgeons could identify critical areas with reduced or increased blood flow, helping to prevent complications such as anastomotic leakage. In our overview, nine papers focused on perfusion or saturation imaging of vasculature or tissue.^44^^,^^45^^,^^48^^,^^52^^,^^55^^,^^56^^,^^59^^,^^63^^,^^65^ For example, Ayala et al. ^44^^,^^45^ showed the potential of HSI for *in vivo* visualization of perfusion during MIS in partial nephrectomy in patients, indicating that HSI is capable of distinguishing different perfusion states during surgery.

Besides the oxygenation and amount of blood to identify tissue changes, the other most common application of HSI and MSI is the detection and classification of specific tissue types during surgery. In our overview, 11 papers focused on identifying or segmenting a tissue type(s).^46^^,^^50^^,^^51^^,^^53^^,^^54^^,^^60^^,^^68^^,^^69^^,^^71^^,^^73^^,^^74^ For instance, Bali et al.^46^ showed tumor detection in head and neck tumor surgery; the TIVITA™ Mini is used to successfully identify tumor areas. Similarly, in colorectal cancer, HSI is employed for the development of a contactless endoscopic diagnostic support system.^53^ The capabilities of HSI extend to differentiating cancer tissue in the esophagus, stomach, and larynx.^83^ Furthermore, HSI is utilized for the evaluation of organ viability during transplantation procedures, such as uterine transplants in rabbits and sheep, as performed in Ref. 48. Finally, research is being conducted on the use of multispectral imaging combined with 3D endoscopy for surgical guidance.^71^

An important finding is the feasibility of HSI and MSI in various surgical contexts. Studies have demonstrated successful use of HSI during esophagectomies, in the detection of laryngeal tumors, and in evaluating organ viability during transplantation, indicating that the technique could be applied in a wide variety of surgical contexts. Furthermore, these studies show the ability of an HSI system to be integrated into surgical workflows. In some cases, commercial systems are used, thus applying general HSI/MSI, whereas in some cases, specialized systems are developed for a specific application. As such, some papers discussed the technical development of these HSI and MSI systems, where the focus of the paper is the description of the developed system more than the utilization of the system.

#### 3.4.1 Quantitative performance and comparative analysis

In Table 3, the quantitative results of the studies performing segmentation or classification are summarized, including parameters such as the number of participants (N), median age, sensitivity, specificity, accuracy, and AUC. As not all studies report all these parameters, some cells in the table are left blank. It should be noted that only a small number of the total studies performed segmentation or classification, with only seven papers reporting qualitative results on segmentation or classification ability.

**Table 3:** Overview of quantitative results or derived quantitative results from selected studies, where N is the number of participants. Please note that the mentioned studies had significantly differing goals; therefore, direct comparison between these metrics is not possible nor useful.

Study	N	Median age	Sensitivity (%)	Specificity (%)	Accuracy (%)	AUC	Application
Ayala et al. (2023)^45^	10	56	90^b^	90^b^	Not given	0.93^b^	Perfusion assessment
Bali et al. (2024)^46^	12	Not given	72	84	79	Not given	Head and neck tumors
Fukushima et al. (2024)^51^^b^	2	Not given	45	91	89	Not given	Nerve detection
Han et al. (2016)^53^	12	Not given	97	91	94	Not given	Colorectal tumors
Hohmann et al. (2017)^54^	14	67.5^b^	63	64	Not given	70^b^	Stomach tumors
Kumashiro et al. (2016)^59^	21/24^a^	68/65	73/100^a^	82/100^a^	79/100^a^	Not given	Colorectal tumors
Takamatsu et al. (2021)^69^^b^	9	Not given	54	90	88	Not given	Mouse tumor models

a *Ex-vivo* data.

b Estimated from reported results.

c Animal model.

This smaller number of studies that present qualitative results does illustrate the potential of HSI and MSI imaging for MIS. A common aspect of these studies is the focus on monitoring perfusion and ischaemia during surgical procedures; this would enable surgeons to identify areas with reduced blood flow by measuring oxygen saturation and blood volume in tissues. This additional information could lead to better decision-making during surgeries and help prevent complications such as anastomotic leakage. Furthermore, some studies demonstrated that HSI and MSI are effective in distinguishing tumor tissue from healthy tissue. For example, studies on head and neck tumors showed that HSI systems could detect tumor locations.^46^ Similarly, in colorectal cancer, HSI has been shown to assist in tumor identification.^53^^,^^69^

In studies on laparoscopic surgery, it was shown that a handheld hyperspectral camera produced qualitatively comparable parameter images to those from systems used in open surgery. The image quality was sufficient to help the surgeon determine the most suitable location for the anastomosis. Although there were quantitative discrepancies between the systems, these were attributed to technical differences rather than study design issues.

For example, Ilgen et al.^55^ compared an MIS-HSI system to an approved HSI system for open surgery, the TIVITA^®^ Tissue, where they compared derived tissue parameter maps using a mean absolute error (MAE) metric. Two groups are created, where an acquisition is made with both the open system (N=30) and the MIS system (N=21); here, they find an MAE as high as 12.6±8.8 for near-infrared perfusion index (NIR-PI), and as low as 2.6±4.4 for tissue water index (TWI). A possible explanation they provide for differences and possible problems for implementation of the MIS system compared with the open is the difficulty in maintaining distance and stability with the MIS system, which is not a problem for the open system. Similarly, Kohler et al.^57^ compared a high-resolution system they developed to the same approved open surgery as Ilgen et al. (2024), using a ColorChecker card. Here, they found an RMSE over 11 patches of 0.03±0.02. Furthermore, when comparing the system for a surgical case, the RMSE was 0.02 for the two main absorbance spectra and 0.09±0.05 for eight randomly selected ROIs. This shows a good overlap in the spectra between the open system and the MIS system.

Similar to the previously discussed studies, Pfahl et al.^64^ compared their developed system to the TIVITA^®^ Tissue, where they again calculated the MAE and RMSE between the two systems. The average MAE-values for the tissue parameter maps varied among the different parameters, with the parameter for perfusion from deeper tissue (NIR-PI) having an MAE of 14±3, and TWI being 10±2, which is higher than what was reported by Ilgen et al. (2024).^55^ The average RMSE between the spectra for both systems was 0.1±0.03 from 500 to 750 nm and 0.15±0.06 from 750 to 1000 nm, being higher than what Kohler et al.^57^ reported for their system. Here, it is also good to note that Pfahl et al.^64^ also reported the intra-system RMSE for both setups, which was equal for both systems around 0.05. To summarize the results from Pfahl et al. (2022) and Ilgen et al (2024), both studies showed that when creating a laparoscopic setup, the resulting spectra, and thus any data derived from this, will differ; however, this difference is small enough that the results obtained with an open- or benchtop-HSI system could be transferred into a minimally invasive setup.

Although the performances from the studies are not to be compared directly, five papers focused on cancer detection, where Kumashiro et al. (2016) and Han et al. (2016) both focused on colorectal tumors. Furthermore, when comparing the setups, both the newer setups from Ayala et al.,^45^ Takamatsu et al.,^69^ and Fukushima et al. (2024) show implementation of newer technologies. Ayala et al.^45^ utilized a snapshot camera, which has become more accessible and contains more bands compared with earlier research. Similarly, Takamatsu et al.^69^ and Fukushima et al.^51^ utilized InGaS sensor technology to allow for near-infrared imaging up to 1400 nm, whereas Han et al.^53^ focused on a wavelength range between 405 and 655 nm. Finally, Bali et al.^46^ noted that the studies by Pfahl et al.,^64^ Pfahl et al.,^65^ Thomaßen et al.,^70^ and Ilgen et al.^55^ primarily focused on comparing systems, assessing methodological feasibility, and analyzing tissue parameters such as tissue oxygen saturation (${\mathrm{StO}}_{2}$), NIR-PI, TWI, and oxygenation and hemoglobin index (OHI). They did not provide sensitivity, specificity, or accuracy results. Similarly, Leitner et al.,^60^ Regeling et al.,^83^ Pruitt et al.,^66^ Pruitt et al.,^80^ Yoon et al.,^72^ and Zhang et al.^74^ did not directly report numerical values for these parameters but described successful tissue classifications, and thus, these were not included in the table.

The reviewed studies demonstrate that HSI and MSI can provide clinically relevant information in minimally invasive surgical settings, particularly for perfusion monitoring and tissue or tumor classification, which are the most common applications. Quantitative comparisons between MIS-HSI systems and established open-surgery systems indicate that derived tissue parameters, such as ${\mathrm{StO}}_{2}$, NIR-PI, TWI, and OHI, show good agreement, with only minor spectral differences that do not substantially affect interpretation. Snapshot cameras and advanced sensors such as InGaAs detectors have enabled extended spectral coverage and improved resolution, supporting accurate tissue assessment. Although the number of studies reporting quantitative performance metrics is still limited, the available results confirm the potential of these systems to inform intraoperative decision-making and support real-time evaluation of tissue viability and pathology. However, including more standardized metrics could help the implementation of HSI in the surgical context, especially for regulatory and comparisons between systems; this is a missing factor in the current literature.

Hyperspectral imaging in laparoscopy offers a unique set of advantages compared with conventional imaging modalities used in minimally invasive surgery. Unlike standard (white-light) laparoscopy, which relies on the surgeon’s subjective visual assessment and limited contrast between different tissue types and structures, HSI provides the ability to extract objective, quantitative data on tissue oxygenation, perfusion, and metabolic state through the extraction of optical parameters from the spectral information or through the use of deep-learning-based approaches. This allows for a more informed evaluation of tissue viability and potential complications, particularly in critical decisions such as anastomotic site selection or tumor margin delineation.

When compared with narrow-band imaging (NBI), which uses fixed illumination at two or three specified wavelengths (typically 415, 540, and optionally 600 nm), HSI stands out by offering the ability to select from dozens to hundreds of wavelength bands.^86^ This increased spectral resolution enables more precise tissue classification and improved tumor detection by targeting wavelength bands optimized for specific tissue characteristics. Furthermore, more specialized wavelength bands can be chosen depending on the contrast that is to be visualized, whereas NBI utilizes only a specific set of wavelengths chosen to work for the widest range of applications.

Relative to fluorescence imaging (FI) using exogenous probes, such as indocyanine green (ICG), HSI provides comparable, if not better, assessments of tissue perfusion but with two key advantages: being label-free, no exogenous contrast agents are required, and besides perfusion, saturation can also be measured.^45^ This not only reduces procedural complexity and risks associated with dyes but also allows continuous imaging without concerns about dye clearance or dosing. Moreover, HSI can extract a wider range of physiological parameters, such as oxygen saturation, water content, and hemoglobin concentration, enhancing intraoperative decision-making.^87^ Relative to FI with exogenous probes, HSI can image much deeper due to the wide range of absorbing chromophores, with the penetration depth of the light from the fluorescent dye being much smaller compared with HSI imaging.^88^

Other imaging techniques offer complementary strengths but lack the full functionality that HSI offers. For example, structured light laparoscopy enables 3D surface mapping and blood volume overlays but does not provide spectrally resolved functional data.^89^ Optical coherence tomography (OCT) delivers high-resolution depth imaging but is limited in surface-wide tissue characterization.^90^ Virtual chromoendoscopy, which enhances mucosal patterns using software-based color manipulation, may benefit from HSI as a data source but does not independently offer spectral analysis.^91^ Therefore, the development of HSI/MSI laparoscopes could further be advanced in combination with other techniques. Furthermore, as discussed in the introduction, a larger proportion of MIS is shifting from laparoscopic to robotic surgery. With this shift, the use of hyperspectral cameras in combination with surgical robots could be a key opportunity to implement this technique in an impactful way across a wide range of surgeries. However, this integration has not yet been realized likely due to the technical and regulatory complexity associated with modifying or adding hardware components to certified surgical robotic systems.

### 3.5 Limitations and Future Directions

The aim of this paper was to provide insights and perspectives into hyperspectral/multispectral laparoscopy; however, several limitations warrant further investigation. The scope of the literature search, constrained by HSI and MSI in endoscopy and laparoscope for the last 10 years, may have led to the exclusion of relevant studies. Furthermore, the descriptive analysis of the papers does not fully reconcile conflicting findings nor does it give insight into the study qualities.

## 4 Conclusion

Hyperspectral minimally invasive imaging is an expanding field with several devices and applications being studied. Most studies are classified under T1 and T2, reflecting the early stages of HSI research and its transition to clinical applications, but no studies have reached the level of late-stage clinical trials. The focus remains on emerging imaging techniques rather than large-scale clinical implementation. Current findings indicate the potential of the technique for a variety of intraoperative applications, including blood oxygen measurements for anastomosis viability evaluation, nerve detection using SWIR wavelengths, and tumor identification. Most of the reviewed papers focused on the visible range of the spectra, with most devices operating between 450 and 650 nm, which are well suited for perfusion and oxygenation assessment, with a few systems extending toward 1000 nm to capture contributions from water and lipids. Depending on the application, either spectral scanning or line-scanning devices are most common, in combination with either a Xenon or LED lightsource depending on range and application.

However, these observations also highlight the need for further development toward standardized methodologies, broader spectral coverage for specific applications, and validation in clinical environments. Integrating optimized illumination, high-resolution detectors, and workflow-compatible laparoscopic designs will be key to generating reproducible and clinically actionable data. These steps are needed to further the implementation of HSI-MIS into more advanced implementation tiers, which are not seen currently. Addressing these aspects could help define how optical imaging technologies, such as hyperspectral laparoscopy, can meet unmet clinical needs and guide future progress in surgical imaging.

## Data Availability

Data sharing is not applicable to this article as no new data were created or analyzed.
